# Changes in treatment outcomes in patients undergoing an integrated psychosomatic inpatient treatment: Results from a cohort study

**DOI:** 10.3389/fpsyt.2022.964879

**Published:** 2022-08-25

**Authors:** Monika Sadlonova, Julia Katharina Löser, Christopher M. Celano, Christina Kleiber, Daniel Broschmann, Christoph Herrmann-Lingen

**Affiliations:** ^1^Department of Psychosomatic Medicine and Psychotherapy, University of Göttingen Medical Center, Göttingen, Germany; ^2^Department of Cardiovascular and Thoracic Surgery, University of Göttingen Medical Center, Göttingen, Germany; ^3^German Center for Cardiovascular Research, Göttingen, Germany; ^4^Department of Psychiatry, Massachusetts General Hospital, Boston, MA, United States; ^5^Department of Psychiatry, Harvard Medical School, Boston, MA, United States; ^6^Department of Geriatrics and Early Rehabilitation, St. Joseph-Stift Hospital, Bremen, Germany; ^7^Department of Psychosomatic Medicine and Psychotherapy, Medical Center Kassel, Kassel, Germany

**Keywords:** psycho-cardiology, inpatient psychotherapy, psychosomatic medicine, cardiovascular disease, HRQoL

## Abstract

**Objective:**

In Germany, multimodal psychosomatic inpatient treatment can be initiated for patients with substantial mental disorders (e.g., depression, anxiety, somatoform disorders) and comorbid physical disease. However, studies investigating changes in psychological and functional treatment outcomes, and predictors of long-term treatment effects in patients undergoing psychosomatic inpatient treatment are needed.

**Methods:**

This cohort study analyzed 160 patients aged ≥18 who were treated on an integrated psychosomatic inpatient unit at the University of Göttingen Medical Center. Its aim was to analyze changes in psychological and functional outcomes, and to identify predictors of long-term improvements in health-related quality of life (HRQoL) in patients with comorbid mental and physical illness who were undergoing integrated inpatient psychosomatic treatment. Assessments were completed at admission, discharge, and 12- or 24-month follow-up. Outcomes included physical complaints [Giessen Subjective Complaints List (GBB-24)], psychological symptoms [Brief Symptom Inventory (BSI)], and HRQoL [European Quality of Life Questionnaire (EQ-5D)].

**Results:**

One-hundred sixty inpatients were included (mean age = 53.1 ± 12.6; 53.8% female). There were significant, medium- to large-sized improvements in psychological symptoms (BSI-Global Severity Index; *d* = −0.83, *p* < 0.001), physical symptom burden (*d* = −0.94, *p* < 0.001), and HRQoL (*d* = 0.65, *p* < 0.001) from admission to discharge, and significant, small- to medium-sized greater improvements in all psychological outcomes from admission to follow-up (BSI-GSI: *d* = −0.54, p < 0.001; GBB-24 total symptom burden: *d* = −0.39, *p* < 0.001; EQ-5D: *d* = 0.52, *p* < 0.001). Furthermore, better improvement in HRQoL during hospitalization (partial η^2^ = 0.386; *p* < 0.001) was associated with higher HRQoL at follow-up. Finally, intake of antidepressant at discharge was associated with impaired HRQoL at follow-up (η^2^ = 0.053; *p* = 0.03).

**Conclusion:**

There were significant short- and long-term improvements in psychological symptoms, physical complaints, and HRQoL after treatment on an integrated psychosomatic inpatient unit in patients with mental disorders and a comorbid physical disease.

## Introduction

Among individuals with chronic physical diseases, mental disorders are highly prevalent (1–4) and are associated with impaired quality of life (QoL) (3, 5–7), high disability burden (3, 4, 7, 8), medical complications (7), high healthcare utilization and costs (3, 9), and premature mortality (3, 10). Specifically, mental disorders have been linked to adverse cardiovascular outcomes (e.g., major adverse cardiac events or mortality in coronary heart disease patients) (11, 12), and patients with cardiovascular diseases require complex emotional-cognitive adaptations (11). Despite these relationships, mental disorders are underdiagnosed and undertreated in primary care settings (13), with previous studies demonstrating that over 50% of patients with mental disorders do not receive guideline-concordant treatment that can impact both psychological (e.g., QoL, psychological distress) and medical outcomes (e.g., hospitalizations, disease-related complications, mortality) (7, 14–19). Furthermore, existing treatments rarely utilize a holistic approach that targets both mental and physical symptoms and is guided by psychosomatic principles (20).

One potential treatment option for more severely ill patients with mental disorders (e.g., depression, anxiety, somatoform disorders) and comorbid physical illness is multimodal care in an inpatient psychosomatic medicine setting (21–23). Inpatient psychosomatic care involves inpatient hospitalization for a period of 4–6 weeks, during which patients engage in a variety of therapeutic interventions, including psychotherapy, patient-centered nursing, creative therapies, body-oriented and physical therapy, and pharmacotherapy (22). In Germany, psychosomatic medicine is not a subspecialty of psychiatry but represents a specialized medical discipline, and its clinical core competency is an integrated care approach that focuses specifically on patients with comorbid mental and concomitant physical disease (e.g., psycho-cardiology) (22, 24). As integrative patient-centered care concepts (e.g., psycho-cardiology) are increasingly recommended (24), the present study describes a unique, inpatient psychosomatic setting mainly focused on patients with mental disorders and comorbid cardiovascular diseases (e.g., coronary heart disease, malignant cardiac arrhythmias, heart failure). This setting allows the integration of psychological treatments, including psychological, creative, body-oriented, and specialized cardiac-based treatments, into an Academic Heart Center.

In addition, physical diseases (e.g., diabetes, inflammatory bowel disease, migraine) with pronounced psychological components are treated. A meta-analysis of randomized controlled trials (RCTs) evaluating these types of psychotherapy-focused hospital treatments (25) found that these interventions led to a medium within-group effect size (Hedges’ g = 0.72) improvement in symptoms from admission to discharge, with a small reduction of the effect to follow-up (g = 0.61). However, fewer than 50% of patients in the reviewed studies had comorbid physical diseases and most were under 50 years of age. Therefore, additional research studies investigating short- and long-term treatment effects of multimodal psychosomatic inpatient care in older patients with both mental and physical illnesses are necessary. This would improve the scientific evidence for treatment effects of integrated psychosomatic units in medical centers (e.g., heart centers or internal medicine departments).

Additionally, identifying predictors of response to psychosomatic treatment may be beneficial. To date, both sociodemographic and treatment characteristics have been linked to treatment response, though the existing literature has been mixed. For example, while one cohort study found younger age to be associated with subjective change three months after psychosomatic hospitalization (26), a second observational study (*N* = 1829) found age not be associated with changes in psychological outcomes at admission, discharge, and 1-year follow-up (27). Similarly, though a RCT (*N* = 298) of standardized five-week multimodal cognitive-behavioral therapy (CBT) demonstrated that women with depressive disorders and chronic pain syndromes benefit significantly more than men from the program (28), white men responded more robustly to a CBT intervention than other subgroups in a different study (*N* = 2481) investigating a treatment effects of CBT in patients with minor or major depression after myocardial infarction (29). Furthermore, comorbid physical diseases, lower self-efficacy, and the number of mental disorders were found to be predictors of treatment outcomes (e.g., subjective complaints and negative mood) assessed at 1-year follow-up after inpatient psychosomatic treatment (27). Finally, participation in a targeted outpatient aftercare intervention led to increased long-term effectiveness of inpatient psychosomatic treatment (30). Given the paucity of data, better understanding of predictors for short- and long-term treatment outcomes (e.g., psychological and functional symptom complaints, HRQoL) of integrated inpatient psychosomatic treatments (e.g., psycho-cardiology or psychosomatic internal medicine) in patients with mental disorders and comorbid physical disease is necessary.

To address the above-mentioned gaps, we performed a cohort study to examine changes in psychological and functional outcomes in patients undergoing an integrated, multimodal inpatient psychosomatic treatment program in an academic Heart Center, as well as to identify predictors of HRQoL at follow-up. Given previous scientific findings, we hypothesized that (1) the multimodal, inpatient psychosomatic treatment would lead to significant improvements in psychological and functional outcomes at discharge and 12- or 24-month follow-up compared to admission. Furthermore, we expected that (2) female sex, higher age, and the presence of comorbid cardiovascular disease would moderate these effects; and that (3) both sociodemographic and treatment-related variables (e.g., outpatient psychotherapy, intake of antidepressants) would be associated with HRQoL in this population.

## Materials and methods

### Study design

The primary aim of this single-center cohort study was to assess changes in psychological and functional outcomes from admission to discharge and 12- or 24-month follow-up in patients with mental disorders and a comorbid physical disease undergoing an integrated, inpatient psychosomatic treatment program. The secondary aims were to investigate whether (i) age, sex, and the presence of comorbid cardiovascular disease act as moderators of changes in treatment outcomes; and whether (ii) there are influencing factors on HRQoL at follow-up in patients with indication for an inpatient psychosomatic care with a comorbid physical disease. Ethical approval for this study protocol was obtained from the Ethics Committee of the University of Göttingen Medical Center (#1/10/11) on January 31, 2012.

### Study setting and participants

In total, 160 patients aged 18 years or older who were treated on the multimodal inpatient psychosomatic unit of the Department of Psychosomatic Medicine and Psychotherapy located in the Heart Center of the University Medical Center in Göttingen, Germany, between February 2010 and January 2012 were included. The inclusion criteria were as follows: (a) completed inpatient treatment for at least three weeks; (b) age 18 years or more; and (c) sufficient cognitive skills and ability to speak, read and understand German; The exclusion criteria were severe cognitive impairment, communicative difficulties (e.g., aphasia), or inability to provide informed consent.

Data at baseline and discharge were obtained during routine diagnostics and quality assurance and were extracted from participant records retrospectively. They included detailed medical history data obtained during hospitalization, as well as a set of psychological and functional questionnaires completed upon admission and before discharge. For the prospective follow-up assessment at 12- or 24-month post-discharge (depending on the year of hospitalization), informed consent was obtained from all participants after providing detailed study information. Follow-up data were prospectively collected by mailing the study participants a set of the same questionnaires that had been used during hospitalization and a number of tailored items related to events and treatments that occurred after initial hospital discharge.

### Inpatient psychosomatic treatment

The inpatient psychosomatic unit is run by the Department of Psychosomatic Medicine and Psychotherapy in collaboration with the Department of Cardiology of the University Medical Center Göttingen, Germany, and focuses on treating patients with mental disorders and comorbid cardiovascular or other physical diseases. The unit is integrated into the Göttingen Heart Center and has a treatment capacity for 18 inpatients.

Admission is electively planned after a detailed outpatient interview. The minimum age for admission is 18 years. A maximum age is not defined, but the patients should be physically and psychologically able to participate as much as possible in the treatment setting. Furthermore, common admission criteria for an inpatient stay are the simultaneous occurrence of a mental disorder (e.g., depression, anxiety or somatoform disorder), and a physical disease (e.g., cardiac disease) or severe functional somatic symptoms attributed to a putative underlying medical illness.

The treatment concept of the inpatient unit is based on a bio-psycho-social model (22). The four- to six-week psychosomatic inpatient care is a multimodal treatment combining individual and group psychotherapy (based on psychodynamic psychotherapy and CBT), psychoeducation, art therapy, relaxation training (e.g., progressive muscle relaxation), and body-oriented and physical therapy, including exercise training. Interpersonal and psychosocial problems can be addressed through inclusion of partners or family, as well as through the use of psychosocial skills training. Furthermore, patients receive daily medical visits (e.g., with medical workup or specialized consultations if indicated). If necessary, drug treatment for physical and mental conditions is initiated or adjusted. Each patient receives weekly therapy plans for the duration of their inpatient stay, which determines the daily structure and therapeutic program. In particular, cardiovascular diseases such as coronary heart disease, malignant cardiac arrhythmias, heart failure, and arterial hypertension are treated on the unit, if these are essentially caused by psychological and behavioral factors (including non-adherence) or if they are accompanied by depression, anxiety disorders or post-traumatic stress disorder. In addition, physical diseases (e.g., diabetes, inflammatory bowel disease, migraine) with pronounced psychological components are treated. Finally, specific behavioral treatment components are available for the management of a spectrum of eating disorders (from anorexia nervosa with body mass index <15 kg/m^2^ to severe obesity).

### Study assessments

Participants’ health records were reviewed to obtain sociodemographic and clinical data, including primary admission diagnoses and healthcare utilization. Psychological and functional outcomes were assessed using valid and reliable questionnaires (31–34).

The Giessen Subjective Complaints List (GBB-24) is a 24-item questionnaire for the assessment of physical complaints. For each item there are possible answers from 0 = “not at all” up to 4 = “very much” (31). The individual complaints can be aggregated on four subscales: exhaustion, gastrointestinal complaints, musculoskeletal complaints, cardiovascular complaints. Total symptom burden ranges from 0 to 96.

The Brief Symptom Inventory (BSI) (32) assesses psychopathological and psychological symptoms. The questionnaire consists of 53 items, which are answered using a 5-point Likert scale from 0 = “not at all” to 4 = “very much”. It covers nine symptom dimensions: somatization, obsession-compulsion, interpersonal sensitivity, depression, anxiety, hostility, phobic anxiety, paranoid ideation and psychoticism. A global index of distress called Global Severity Index (GSI) can be created and ranges from 0 to 4.

The EQ-5D (European Quality of Life Questionnaire) is a short instrument, consisting of 5 items, which is used to record health-related quality of life (HRQoL). The 5 items represent the dimensions of mobility, self-care, usual activities, pain/physical complaints and fear/depression (33, 35). Individual item values were transformed into an index score on a scale from 0 to 100, with higher scores indicating better HRQoL.

Self-efficacy was assessed using a short form of the General Self-Efficacy Scale (GSE-6) (34). In the 6-item version, participants are asked to rate the degree to which items applies to them on a scale ranging from 1 “does not apply at all” and the value 4 means “applies completely”. The mean value is created by dividing the sum of all 6 items by the number of items.

### Statistical analysis

Clinical and psychological data are shown as means (M) and standard deviations (SD) of continuous variables or frequencies and percentages of categorical variables. To analyze relationships between physical and psychological variables at all three timepoints and the comorbid physical disorders, Mann-Whitney U tests were performed. To assess differences between the individuals who did or did not complete all three measurements, we performed one-way analyses of variance (ANOVAs) for continuous variables and Fisher’s exact tests for categorical variables.

To examine changes in treatment outcomes of integrated inpatient psychosomatic care, the mean values of the psychological outcomes at admission, discharge, and 12- or 24-month follow-up were compared using ANOVA with repeated measurements. As there were no differences between the subgroups followed for 12 vs. 24 months, both groups were combined for further analyses. The effect sizes were determined either as Cohen’s *d* (*d* ≥ 0.2 as small effect; *d* ≥ 0.5 medium effect; *d* ≥ 0.8 as large effect) or in the form of the partial eta square (η^2^): Concretely, η^2^ ≥ 0.01 assumes a small effect, η^2^ ≥ 0.06 a medium effect, and η^2^ ≥ 0.14 a large effect (36). Medium effect sizes are considered to be clinically relevant. To evaluate the analysis of variance, sphericity was first tested using the Mauchly test. If this could not be accepted, the Greenhouse-Geisser correction was used for the interpretation (37). The intra-subject effects of the two-stage time factor were tested.

To test influencing factors on the change in psychological and functional outcomes over the course of time between admission and discharge, multi-factorial ANOVA with repeated measurements were used. The time of assessment (discharge vs. admission) was defined as the intra-subject factor, and the influencing variable to be analyzed was the between-subject factor. Significant between-subject effects and within-subject contrasts were examined for their effect size using the η^2^. We focused on the evaluation of the interaction term (time*between-subject factor) in order to test the influence of the between-subject factor on the temporal development of the individual psychometric findings.

Finally, we used Spearman’s correlation analysis to examine bivariate relationships between HRQoL at follow-up and HRQoL at admission and discharge, as well as intake of antidepressants at discharge, referral for outpatient psychotherapy post-discharge, and self-efficacy at admission and discharge. Afterward, a multi-factorial ANOVA with repeated measurements was performed to analyze factors associated with change in HRQoL over the course of time between follow-up and admission. The time of assessment (follow-up vs. admission) was defined as the intra-subject factor, and the influencing variable to be analyzed was the between-subject factor. Significant between-subject effects and within-subject contrasts were examined for their effect size using η^2^. To compare mean scores in psychological and functional variables between patients with and without intake of antidepressants, a paired t-test was performed.

All analyses were performed using SPSS software, version 27 (SPSS Inc., Chicago, IL, United States). A *p* value of <0.05 was considered statistically significant.

## Results

### Recruitment and baseline characteristics

**FIGURE 1:**
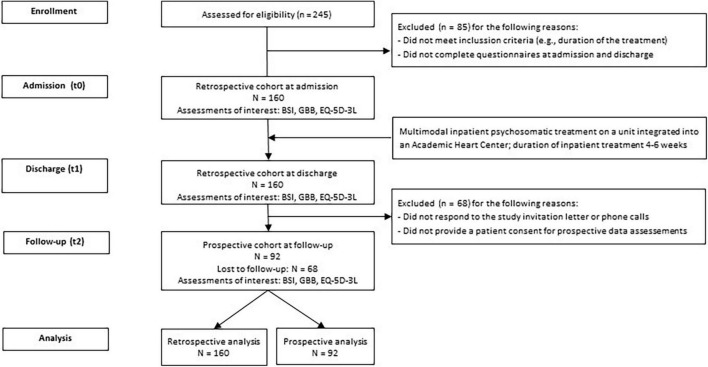
Flow chart of the study.

We identified 245 eligible patients who underwent inpatient psychosomatic treatment between February 2010 and January 2012. Of these, 160 patients (mean age in years = 53.1, SD = 12.6; 53.8% female) completed both the admission and discharge questionnaires and were enrolled in the study. Of these, 92 patients (mean age = 55.6, SD = 12.4; 52.2% female) completed the 12- or 24-month follow-up questionnaires (Figure 1). The clinical and psychological baseline characteristics of the total sample are shown in Table 1. In the total sample, the most prevalent mental disorders were affective disorders (38.1%), followed by somatoform disorders (30%), and anxiety and obsessive-compulsive disorders (19.4%). The most common physical comorbidities were hypertension (50%), ischemic coronary heart disease (20.6%), diabetes (10.6%), and atrial fibrillation/flutter (8.1%). In Whitney–Mann *U* tests, there were no associations between the comorbid physical diseases and the physical or psychological outcomes at any of the three timepoints.

**TABLE 1:** Characteristics of clinical and psychological variables.

	Total sample (N = 160)
Variables (range)	Mean ± SD or %
Women	53.8%
Age (years) (range 22–83)	53.1 ± 12.6
**Main diagnosis**	
F30-39 Affective disorders	38.1%
F40-42 Anxiety and OCD	19.4%
F43 PTSD and ASD	3.1%
F45 Somatoform disorders	30.0%
F50 Eating disorders	3.1%
F54 Psychological and behavioral factors associated with disorders or diseases	3.1%
Others	3.1%
**Comorbid physical diseases**	
Hypertension	50.0%
Coronary heart disease	20.6%
Hyperlipidemia	20.0%
Diabetes mellitus	10.6%
Atrial fibrillation/flutter	8.1%
Other cardiac diseases History of stroke	18.3% 3.1%
Hypothyroidism	6.25%
Psoriasis	3.8%
Migraine	3.1%
Number of comorbid mental disorders (range 0–4)	2.0 ± 1.1
Number of comorbid physical diagnoses (range 0–10)	2.1 ± 2.1
BSI-GSI (range 0–4*)	1.1 ± 0.6
GBB total symptom burden (range 0–96*)	37.3 ± 15.0
GSE-6 (range 1–4*)	1.6 ± 0.7
EQ-5D index (range 0–100*)	53.9 ± 16.4
Duration of inpatient treatment (days; range 23–59 days)	40.9 ± 5.6

ASD, acute stress disorder; BSI, Brief Symptom Inventory; EQ, European Quality of Life Questionnaire; GBB, Giessen Subjective Complaints List; GSI, Global Severity Index; GSE, General Self-Efficacy Scale; OCD, obsessive-compulsive disorder; PTSD, posttraumatic stress disorder; SD, standard deviation. *Range of the assessment.

Patients lost from discharge to follow-up (*N* = 68) were comparable to completers of all measurements in all variables at follow-up (*N* = 92), aside from having lower age (completers: mean age = 55.6, SD = 12.4; dropout: mean age = 49.7, SD = 12.1; *p* = 0.003), and higher psychological distress (BSI-GSI; completers: 1.0 ± 0.6; dropout: 1.2 ± 0.6; *p* = 0.004).

### Changes in psychological and functional treatment outcomes

Regarding psychological and functional outcomes (Table 2), there were significant, large-sized improvements in psychological symptoms (BSI-GSI: paired *t*-test: M = −0.45; SD = 0.54; effect size: *d* = −0.83; 95% CI −0.53, −0.36; *p* < 0.001) and physical complaints (GBB total symptom burden: M = −11.74; SD = 12.44; *d* = −0.94, 95% CI −13.68, −9.80; *p* < 0.001), and significant medium-sized improvements in HRQoL (EQ-5D: M = 10.77, SD = 16.70; *d* = 0.65, 95% CI 8.10, 13.45, *p* < 0.001), from admission to discharge in patients undergoing integrated inpatient psychosomatic treatment. The adjusted general linear models (Supplementary Files 1, 2) found no significant moderating effects of age, sex, and comorbid cardiovascular diagnosis on changes in physical and psychological symptoms from admission to discharge. Changes in HRQoL at discharge compared to admission were not significant after full adjustment for sex, age, and cardiac diagnosis, and the included variables did not show a significant moderating effect on HRQoL.

**TABLE 2:** Changes in psychological scale scores at discharge compared to admission (paired *t*-tests, *N* = 160).

Outcome variables	Admission	Discharge	Mean difference (discharge-admission) (*N* = 160)						
	*M* (SD)	*M* (SD)	*M*	SD	95 CI%	T	df	*P*	*d* *
BSI-GSI	1.08 (0.60)	0.63 (0.48)	−0.45	0.54	−0.53, −0.36	10.43	159	<0.001	−0.83
GBB total symptom burden	37.30 (0.48)	25.56 (15.24)	−11.74	12.44	−13.68, −9.80	11.94	159	<0.001	−0.94
EQ-5D-3L score	54.07 (16.37)	64.84 (16.97)	10.77	16.70	8.10, 13.45	−7.96	151	<0.001	0.65

BSI, Brief Symptom Inventory; df, degree of freedom; EQ, European Quality of Life Questionnaire; GBB, Giessen Subjective Complaints List; GSI, Global Severity Index; M, mean; SD, standard deviation; *P*, significance level. *P* value of <0.05 was considered statistically significant; *d = Cohen’s *d* (*d* ≥ 0.2 as small effect; *d* ≥ 0.5 medium effect; *d* ≥ 0.8 as large effect).

**FIGURE 2:**
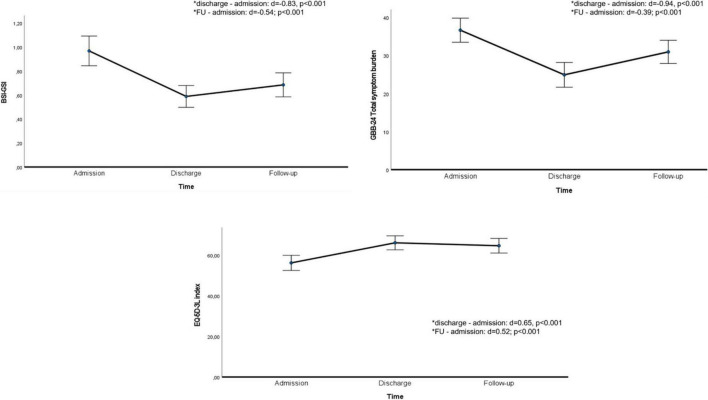
Time course of BSI-GSI, GBB-24, and EQ-5D variables. BSI, Brief Symptom Inventory; CI, confidence interval; EQ, European Quality of Life Questionnaire; FU, follow-up; GBB, Giessen Subjective Complaints List; GSI, Global Severity Index. Asterisks (*) indicate significant differences between adjacent time points. Error bars indicate 95% CIs.

At follow-up (Table 3 and Figure 2), patients still showed significant, small- to medium-sized improvements in psychological symptoms (BSI-GSI: M difference [FU - admission] = −0.283, 95% CI −0.40, −0.16; *d* = −0.54; *p* < 0.001), physical symptoms (GBB total symptom burden: M difference = −5.705, 95% CI −8.97, −2.44; *d* = −0.39; *p* < 0.001), and HRQoL (EQ-5D: M difference = 8.441, 95% CI 4.61,12.28; *d* = 0.52; *p* < 0.001) compared to admission. Between discharge and follow-up there was only a small and non-significant increase in psychological symptoms and only minimal decrease in HRQoL. Physical symptoms increased again at follow-up compared to discharge (M difference [FU - discharge] = 6.031, 95% CI 2.584, 9.477; *d* = 0.40; *p* < 0.001) but remained significantly lower than on admission. Changes in physical and psychological symptoms as well as HRQoL, were unrelated to the timing of follow-up assessment at 12 vs. 24 months.

**TABLE 3:** Changes of psychological outcomes at follow-up compared to admission and discharge (*N* = 92).

Outcomes variables	Admission	Discharge	Follow-up	Mean difference (follow-up - admission)					Mean difference (follow-up - discharge)				
	*M* (SE)	*M* (SE)	*M* (SE)	*M* dif.	SE	95% CI	*P*	*d* *	*M* dif.	SE	95% CI	*P*	*d* *
BSI-GSI	0.97 (0.06)	0.59 (0.05)	0.69 (0.05)	−0.28	0.05	−0.40, −0.16	<0.001	−0.54	0.10	0.05	−0.01, 0.01	0.11	0.20
GBB-24 total symptom burden	36.66 (1.59)	24.92 (1.64)	30.95 (1.54)	−5.71	1.34	−8.97, −2.44	<0.001	−0.39	6.03	1.41	2.58, 9.48	<0.001	0.40
EQ-5D-3L score	56.24 (1.88)	66.12 (17)	64.68 (1.81)	8.44	1.57	4.61, 12.28	<0.001	0.52	−1.44	1.38	−4.82, 1.93	0.90	−0.08

BSI, Brief Symptom Inventory; EQ, European Quality of Life Questionnaire; GBB, Giessen Subjective Complaints List; GSI, Global Severity Index; *M*, mean; M dif., mean difference; SE, standard error; *P*, significance level. *P* value of <0.05 was considered statistically significant; *d = Cohen’s *d* (*d* ≥ 0.2 as small effect; *d* ≥ 0.5 medium effect; *d* ≥ 0.8 as large effect).

In univariate correlation analysis, there was a significant association between EQ-5D-3L at follow-up and EQ-5D-3L at admission (Spearman’s rho = 0.541, *p* < 0.001) and discharge (*r* = 0.648, *p* < 0.001), and a significant negative association between EQ-5D-3L at follow-up and intake of antidepressant at discharge (*r* = −0.067, *p* = 0.021). There were no significant associations between HRQoL at 12- or 24-month follow-up with referral of outpatient psychotherapy after discharge (*r* = −0.067, *p* = 0.529), self-efficacy at admission (*r* = 0.156, *p* = 0.144), or self-efficacy at discharge (*r* = 0.112, *p* = 0.299). In multi-factorial ANOVA with repeated measurements, improvement in HRQoL during hospitalization (partial η^2^ = 0.386, *p* < 0.001) was associated with better HRQoL at follow-up. Furthermore, intake of antidepressant at discharge was associated with impaired HRQoL at follow-up (partial η^2^ = 0.053, *p* = 0.03); there was no significant effect of follow-up assessment performed at 12 vs. 24 months on HRQoL at follow-up (all *p* > 0.05, Table 4). Finally, we performed a paired *t*-test to compare patients with and without intake of antidepressants. Patients with intake of antidepressant showed higher psychological distress (*p* < 0.01) as well as symptom burden (*p* = 0.02) at admission but not at discharge (*p* = 0.06; *p* = 0.23, respectively) compared with patients without a treatment with antidepressant. The HRQoL was only insignificantly worse in patients receiving antidepressants at admission and discharge while a significant difference emerged during follow-up (Supplementary File 2).

**TABLE 4:** Multi-factorial ANOVA with repeated measures showing factors associated with change in HRQoL between follow-up and admission.

	Type III sum of squares	df	Mean square	*F*	*P*	Partial η^2^*
**EQ-5D-3L (follow-up–admission)**						
Time	224.62	1	224.62	3.46	0.06	0.041
Follow-up assessment at 12- or 24-month	49.71	1	49.71	0.12	0.73	0.001
Change in EQ-5D-3L during hospitalization	2867.66	1	2867.66	6.79	0.01	0.077
Intake of antidepressant at discharge	2562.2	1	2562.2	6.07	0.02	0.07
Time*follow-up assessment at 12- or 24-month	3.6	1	3.6	0.06	0.81	0.001
Time*change in EQ-5D-3L during hospitalization	3304.01	1	3304.01	50.85	< 0.001	0.386
Time*intake of antidepressant at discharge	296.17	1	296.17	4.56	0.03	0.053
Error(time)	5263.33	81	64.98	–	–	–

ANOVA, analysis of variance; df, degree of freedom; EQ, European Quality of Life Questionnaire; *P*, significance level. *P* value of <0.05 was considered statistically significant; *η^2^ = Eta (η^2^ ≥ 0.01 assumes a small effect, η^2^ ≥ 0.06 a medium effect, and η^2^ ≥ 0.14 a large effect).

## Discussion

This single-center cohort study demonstrates significant medium- to large-sized improvements in psychological and physical outcomes (BSI, GBB), and HRQoL (EQ-5D) in patients with mental disorders and physical comorbidity treated on an integrated inpatient psychosomatic unit in an academic Heart Center. These improvements were independent of age and sex and for most variables also of cardiovascular comorbidity. Small- to medium-sized improvements in all outcomes were still found at 12- or 24-month follow-up compared to admission. Better improvement of HRQoL during hospitalization was associated with substantially higher HRQoL at follow-up. Continued psychotherapy after discharge or self-efficacy at admission or discharge did not show significant associations with HRQoL at 12- or 24-month follow-up, while antidepressant medication at discharge was independently associated with poorer HRQoL at follow-up.

Inpatient psychosomatic treatment provides a protective environment with a variety of therapeutic interventions (e.g., individual and group psychotherapy, art therapy, body-oriented and physical therapy, pharmacotherapy), and can focus specifically on patients with a comorbid physical disease (e.g., with a cardiac diagnosis) (22). A meta-analysis of RCTs evaluating psychotherapeutic hospital treatments in Germany (25) demonstrated a medium within-group effect size for symptom change at discharge with a small reduction to follow-up, but most patients in this analysis were younger and did not have a significant physical comorbidity. The current analysis extends these findings by demonstrating significant medium- to large-sized improvements in psychological and functional outcomes specifically in patients with a mental disorder and a comorbid physical disease undergoing an integrated inpatient psychosomatic treatment in an academic Heart Center. Furthermore, these treatment effects largely persisted at follow-up, as small- to medium-sized improvements in all outcomes were still found at 12- or 24-month follow-up compared to admission. These findings are of high clinical relevance, as mental disorders are associated with the highest disability burden of all larger disease categories (8), and as the health goals have been shifted to decreasing disability burden rather than only to increasing life expectancy (38).

However, the present study did not compare the changes in mental health outcomes between integrated inpatient psychosomatic treatment with multimodal components (e.g., individual and group psychotherapy, art therapy, relaxation training, body-oriented and physical therapy, and specialized medical care) to an active comparator (e.g., outpatient or inpatient psychotherapy or psychiatric treatment) in patients with physical comorbidities. Future studies are necessary to provide more information on the following aspects in patients with mental health disorder and a comorbid physical disease: (a) effective duration of inpatient treatment (e.g., the presented integrated unit provides treatment for 4–6 weeks); (b) effectiveness of the treatment domains; and (c) cost-effectiveness of integrated multimodal inpatient units with specialized care of physical comorbidities (e.g., psycho-cardiological or psycho-diabetological units) compared to general psychosomatic/psychiatric units. Given the trend of sub-specialization in medicine, it might be of clinical importance that psychosomatic (and psychiatric) inpatient units focus on prevalent physical comorbidities in patients with mental health disorders with further expertise in areas such as psycho-cardiology, psycho-diabetology, or psycho-oncology. However, better scientific evidence is necessary to implement more sub-specialized inpatients units not only in the German health system but also worldwide.

Furthermore, the finding that higher age and a comorbid cardiovascular diagnosis did not impair the improvements observed during and after inpatient treatment highlights the impact of an integrated inpatient psychosomatic care on psychological and functional outcomes in patients with cardiovascular comorbidity (e.g., coronary heart disease, atrial fibrillation/flutter or hypertension) who are typically older than those studies in the previous meta-analysis (25). This is of clinical relevance as mental disorders have been linked to adverse cardiovascular outcomes (e.g., major adverse cardiac events or mortality in coronary heart disease patients) (11, 12), and patients with cardiovascular diseases require complex emotional-cognitive adaptations (11). For example, a study (*N* = 93) comparing an integrated concept of psycho-cardiac rehabilitation vs. monodisciplinary cardiac or psychosomatic rehabilitation showed that cardiac patients benefit more from an integrated psycho-cardiac treatment concept (39). In line with these results and ours, integrative patient-centered care concepts (e.g., psycho-cardiology) are increasingly recommended for treatment of patient with mental disorders and concomitant physical disease (24).

Finally, in the present study, intake of antidepressants at discharge was independently associated with poorer HRQoL at follow-up. There might be several explanations for this finding. First, patients on antidepressants showed higher symptom burden and psychological stress at admission compared with patients without intake of antidepressants. Therefore, higher initial symptom severity of major depressive disorder (MDD) may have been a reason for antidepressant prescription, which would suggest that it is the initial severity of depression rather that the medication itself that impaired follow-up HRQoL scores in patients taking antidepressants. However, our model controlled for baseline HRQoL, making such inverse causation less likely. Previous studies in patients with MDD undergoing inpatient treatment or day hospital treatment (40, 41) showed that intake of antidepressants was significantly related to more severe depression at admission and discharge that can lead to complex treatment situation (e.g., high number of antidepressants, switch in medication) (41). Second, antidepressants might have greater effects on depressive symptoms than on HRQoL. For example, a study investigating relationship between depressive symptoms and HRQoL in inpatients with MDD before and after 6-week treatment with fluoxetine demonstrated that antidepressant treatment was associated with a greater extent of change in depressive symptoms than in HRQoL (42). In our study, however, also the psychological symptoms remained significantly higher in the medicated vs. unmedicated group. Finally, the effect of antidepressants on HRQoL might not be as sustained at 12- or 24-month follow-up. Consistent to this explanation, a recent study in patients with MDD comparing a cohort with and without intake of antidepressants (43) showed that the real-world effect of antidepressant intake does not continue to improve patients’ HRQoL over time, and the effect of antidepressants on improvements of HRQoL was limited to the initial 2–3 months of treatment. However, the HRQoL scores did not sufficiently improve in long-term compared to the general population (44, 45). Longer-term follow-up of randomized, controlled trials of antidepressant treatment efficacy would help to clarify the long-term effects of these medications on both depressive symptoms and HRQoL.

In summary, this cohort study provides further scientific support for significant improvement in patient-reported outcomes after integrated psychosomatic inpatient care and for factors influencing long-term improvements in HRQoL in patients with mental disorder and a concomitant physical disease (e.g., cardiac disease). The strengths of our study are the longitudinal approach with various psychological and functional outcomes at three time points. However, our study has several limitations. Firstly, this is a single-center cohort study without randomization or an active comparator. Secondly, we excluded 68 patients, as they did not complete all the study assessments. Finally, the German system with established departments for psychosomatic medicine and psychotherapy with inpatient treatments of 4–6 weeks duration and more represents a unique treatment system worldwide which impedes generalizability of our results to other health care systems.

## Conclusion

Multimodal, integrated inpatient psychosomatic treatment was associated with significant medium- to large-sized improvements in physical and psychological symptoms, and HRQoL in inpatients with mental disorders and a comorbid physical disease. These results indicate that integrative patient-centered care concepts (e.g., psycho-cardiology) are useful for treatment of patient with somatic-mental comorbidity. Finally, improvement in HRQoL from admission to discharge seems to remain mostly stable over 1–2 years. Large, randomized-controlled, multi-center clinical trials investigating the effectiveness of integrated, specialized (e.g., psycho-cardiology, psycho-diabetology) psychosomatic inpatient treatments compared to an active comparator are needed to confirm their impact on psychological, functional, and medical outcomes. Furthermore, cost-effectiveness analyses should be provided. However, for the multimorbid patients treated on the unit studied here, inpatient treatment is often the last resort after unsuccessful outpatient therapies. Both, ethical considerations and clear patient preferences for an established and probably effective treatment make randomized trials hard to conduct in this setting, as long as no equally attractive treatment of proven efficacy can be offered as a comparator.

## Data availability statement

The dataset generated and/or analyzed for the present article is not available for sharing as the informed consent form did not include information about data sharing policy. Requests to access the datasets should be directed to MS, msadlonova@mgh.harvard.edu.

## Ethics statement

Ethical approval for this study protocol was obtained from the Ethics Committee of the University of Göttingen Medical Center (#1/10/11) on January 31, 2012. The patients/participants provided their written informed consent to participate in this study.

## Author contributions

MS and CH-L: conceptualization, methodology, statistical analyzes, and writing—original draft. JKL and CK: conceptualization, investigation, project administration, and data curation. JKL, CK, CC, DB, and CH-L: writing—review and editing. CC and CH-L: supervision. MS: data curation and writing—review and editing. All authors have read and agreed to the published version of the manuscript.
