# Supplementary material for: Changes in treatment outcomes in patients undergoing an integrated psychosomatic inpatient treatment: Results from a cohort study
Source: Front Psychiatry. 2022 Aug 25;13:964879. doi: 10.3389/fpsyt.2022.964879 (PMC9453315; doi:10.3389/fpsyt.2022.964879)
Supplement: Supplementary file 1 [file Table_1.pdf]

Supplementary File S1. Repeated measures ANOVA showing changes in psychological scales from admission to discharge and effects of sex, age and cardiovascular comorbidity.

	Type III Sum of Squares	df	Mean Square	F	P	Partial η^{2*}
BSI-GSI						
time	1.09	1	1.09	7.44	<0.01	0.046
sex	0.00	1	0.00	0.00	0.96	0.000
age	1.17	1	1.17	2.63	0.11	0.017
cardiac diagnosis	0.24	1	0.24	0.53	0.47	0.003
time*sex	0.32	1	0.32	2.18	0.14	0.014
time*age	0.05	1	0.05	0.38	0.56	0.002
time*cardiac diagnosis	0.09	1	0.09	0.61	0.44	0.004
error(time)	22.81	156	0.15	-	-	-
GBB-24 total symptom burden						
time	347.70	1	347.70	4.52	0.04	0.028
sex	26.47	1	26.47	0.07	0.79	0.000
age	7.36	1	7.36	0.02	0.89	0.000
cardiac diagnosis	762.49	1	762.49	2.01	0.16	0.013
time*sex	0.22	1	0.22	0.00	0.96	0.000
time*age	9.85	1	9.85	0.13	0.72	0.001
time*cardiac diagnosis	186.69	1	189.69	2.43	0.12	0.015
error(time)	12008.89	156	76.98	-	-	-
EQ-5D-3L						
time	437.90	1	437.90	3.09	0.08	0.020
sex	354.28	1	354.28	0.86	0.35	0.006
age	253.46	1	253.46	0.62	0.43	0.004
cardiac diagnosis	1505.10	1	1505.10	3.67	0.06	0.024
time*sex	25.47	1	25.47	0.18	0.67	0.001
time*age	0.10	1	0.10	0.01	0.93	0.000
time*cardiac diagnosis	32.07	1	32.07	0.23	0.64	0.002
error(time)	20964.08	148	141.65	-	-	-

Note. BSI=Brief Symptom Inventory; df=degree of freedom; EQ=European Quality of Life Questionnaire; GBB=Giessen Subjective Complaints List; GSI=Global Severity Index; P=significance level. P value of < 0.05 was considered statistically significant; *η^2=Eta ($\eta 2 \geq 0.01$ assumes a small effect, $\eta 2 \geq 0.06$ a medium effect, and $\eta 2 \geq 0.14$ a large effect).
