# Supplementary material for: Changes in treatment outcomes in patients undergoing an integrated psychosomatic inpatient treatment: Results from a cohort study
Source: Front Psychiatry. 2022 Aug 25;13:964879. doi: 10.3389/fpsyt.2022.964879 (PMC9453315; doi:10.3389/fpsyt.2022.964879)
Supplement: Supplementary file 2 [file Table_2.pdf]

Supplementary File S2. Comparison of BSI-GSI, GBB and EQ-5D-3L scores between patients with and without intake of antidepressants at discharge.

	Intake of antidepressants at discharge (N=58)	No intake of antidepressant at discharge (N=34)	
Variable	M (SD)	M (SD)	p
BSI-GSI at admission	1.1 (0.08)	0.72 (0.09)	<0.01
BSI-GSI at discharge	0.7 (0.5)	0.5 (0.4)	0.06
BSI-GSI at follow-up	0.8 (0.5)	0.5 (0.4)	<0.01
GBB total symptom burden at admission	39.6 (13.9)	31.9 (16.1)	0.02
GBB total symptom burden at discharge	26.4 (15.0)	22.4 (16.3)	0.23
GBB total symptom burden at follow-up	33.7 (13.7)	26.4 (15.4)	0.02
EQ-5D-3L at admission	53.6 (15.6)	59.3 (19.3)	0.13
EQ-5D-3L at discharge	64.0 (15.4)	69.2 (16.1)	0.14
EQ-5D-3L at follow-up	61.1 (16.0)	70.4 (15.4)	<0.01

Note. BSI=Brief Symptom Inventory; EQ=European Quality of Life Questionnaire; GBB=Giessen Subjective Complaints List; GSI=Global Severity Index; M=mean; SD=standard deviation; P=significant nivea. P value of < 0.05 was considered statistically significant.
